# How the Structure and Wettability Properties of *Morpho peleides* Butterfly Wings Can Be a Source of Inspiration

**DOI:** 10.3390/biomimetics10020089

**Published:** 2025-02-03

**Authors:** Louise Burdin, Anne-Catherine Brulez, Radoslaw Mazurczyk, Jean-Louis Leclercq, Stéphane Benayoun

**Affiliations:** 1 Laboratoire de Tribologie et Dynamique des Systèmes, UMR5513, 69130 Ecully, France; anne-catherine.brulez@itech.fr (A.-C.B.); stephane.benayoun@ec-lyon.fr (S.B.); 2 Department of Chemistry, Institut Textile et Chimique|ITECH, 69130 Ecully, France; 3 Institut des Nanotechnologies de Lyon, Université de Lyon, UMR5270, 69130 Ecully, France; radoslaw.mazurczyk@ec-lyon.fr (R.M.); jean-louis.leclercq@ec-lyon.fr (J.-L.L.)

**Keywords:** *Morpho* butterfly, biomimetic, wettability, superhydrophobicity, anisotropic wettability, multiscale roughness

## Abstract

In this study, the wettability of *Morpho peleides (M. peleides)* wings is studied. Using a goniometer, the contact angles of ~136° were measured. Although few studies have provided a general overview of the static wettability properties of *M. peleides* and the particularly anisotropic morphology of their wings, a detailed analysis of wettability properties is proposed. The results indicate anisotropic wettability, with a sliding angle of 7° when the wings were tilted away from the insect’s body and 29° when the wings were tilted toward the insect’s body. The Extrand model, coupled with a hierarchical approach, was also employed to describe the wettability behavior of *M. peleides* wings and its relationship with the dual roughness scale of the wings. Owing to these models, it has been demonstrated that the micrometric scale of the wing structure is primarily responsible for the static wettability properties of the *M. peleides*, while the nanometric scale influences the dynamic wettability of the wing. Moreover, compared to the topography, the wing’s chemistry has very little effect on the wettability properties of the *M. peleides*.

## 1. Introduction

Over the past few years, there has been increasing interest in mimicking nature. Nature has continuously evolved to adapt and ensure survival in a constantly changing environment. Tens of millions of animal and plant species have specific surface functionalities to meet their needs. Therefore, nature is an inexhaustible source of inspiration for researchers, allowing them to manufacture new functional materials. For example, superhydrophobicity is a highly desired property because it can be used in numerous fields. Superhydrophobic surfaces prevent corrosion [1,2,3,4] and frost [5] and have self-cleaning properties [6]. In nature, one of the most famous examples of superhydrophobic surfaces is the lotus leaf. Its dual micro–nanostructure was revealed for the first time by Neinhuis and Bartholtt in 1997 [7]. Another example of a superhydrophobic surface found in nature is the wings of *M. peleides*.

**Figure 1:**
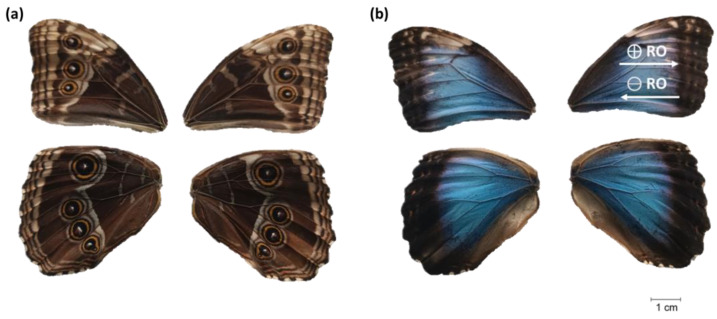
Images of the ventral (**a**) and dorsal wings (**b**) of the *M. peleides*. ⊕RO corresponds to the rolling outward direction. ⊖RO is the direction opposite to the rolling outward direction.

However, the *M. peleides* butterfly is mostly known for its blue metallic shimmer (Figure 1). Only male *Morpho* butterflies display this vibrant color, to communicate with females [8]. Additionally, as shown in Figure 1, only the dorsal part is blue, and the ventral part is brown. This adaptation aids the butterfly in camouflaging and protecting itself from predators upon landing with closed wings [8]. During *Morpho* butterfly flight, it appears as a sporadic presence, and its unpredictable trajectory makes it challenging to anticipate its path [8].

The blue metallic color originates from the periodic, multilayered scales covering the wing’s surface. Each scale is composed of ridged stripes that exhibit similar shapes and are arranged on the scales in an orderly manner, resulting in a high density and strong reflectivity. Moreover, the exact positions of the ridged stripes are also disordered in the subwavelength, thereby creating interference.

This micro–nanostructure that implies a bright structural color has been the subject of scientific curiosity for decades [9,10,11,12,13]. This unique structure can be used in numerous applications such as self-cleaning coatings [14], thermal imaging sensors [15], and efficient solar cells [16].

Nevertheless, wings often exhibit a multitude of intelligent multifunctional properties [17], and, to the best of our knowledge, one of the least studied characteristics of the *Morpho* butterfly is its water repellency in addition to its anisotropic dynamic wettability. Such wettability is directly linked to the butterfly’s survival; water droplets roll in the rolling outwards direction (⊕RO), as shown in Figure 1. This phenomenon not only shields the body but also ensures that the weight of the droplet does not impede its ability to take flight, as well as guaranteeing the cleanliness of the wings. This anisotropy arises from the oriented structures on the surface, which results in specific wettability characteristics depending on the direction [14].

The wettability behavior of a surface can also be explained by two classical models. In the Wenzel model [18], the droplet completely wets the surface, including its asperities. Conversely, in the Cassie–Baxter model [19], the droplet remains on top of the asperities, leaving a layer of air underneath. The Wenzel and Cassie–Baxter models are defined by Equations (1) and (2), respectively:

(1) $$\cos\;\theta_{W}=r\cos\;\theta_{Y}$$

where r is the roughness factor, which is defined as the area of the rough surface divided by the area of an equivalent flat surface.

(2) $$\cos\theta_{\mathrm{CB}}=-1+\varphi s(\cos\theta_{Y+1})$$

θ_Y_ corresponds to the apparent contact angle (CA) on the smooth surface and φs is the area fraction of the solid surface.

These models demonstrate that a droplet can exhibit different behaviors depending on its affinity to the surface. This affinity is directly related to surface roughness. In the case of a superhydrophobic surface, the static CA must exceed 150° and the hysteresis must be lower than 10°. Hysteresis is defined as the difference between the advancing CA and the receding CA [20]. Dettre and Johnson [21] experimentally introduced the influence of roughness on the wettability properties of a surface. They measured the advancing and receding CAs on a hydrophobic wax. Between each measurement, the wax was annealed to reduce its roughness. Thus, when the roughness is high, the hysteresis is low, whereas when the roughness is low, the hysteresis is greater.

Surface chemistry also influences the droplet’s affinity with the surface. In the Wenzel model, the roughness parameter r is always >1. Thus, the roughness amplifies the initial character of the surface. For example, if the surface is initially hydrophilic (θ_Y_ < 90°), the equation implies that hydrophilicity will increase. Conversely, because the area fraction of the solid surface φ_s_ will always be <1, the Cassie–Baxter model will always amplify the hydrophobicity.

In 2002, Extrand [22] was one of the first to propose a model to predict whether a liquid will remain suspended on the asperities of a surface (i.e., Cassie–Baxter state) or will fill these asperities (i.e., Wenzel state). This model is based on two new parameters:

o The contact line density Λ, defined using the following equation:

(3) Λ=pδ

where p is the asperity perimeter and δ is the area density of the asperities.

**Figure 2:**
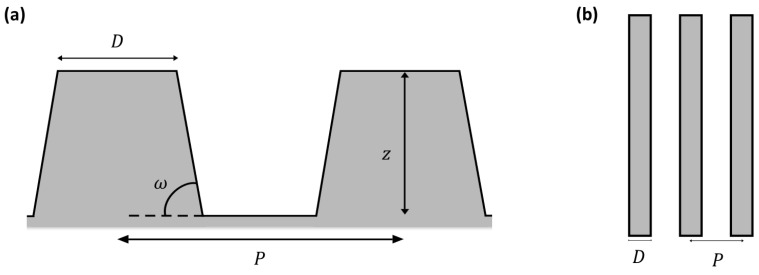
A schematic representation of the stripes seen from the side (**a**) and the top (**b**).

In the case of stripes, p=2P and $\delta =\frac{1}{P^{2}}$, which implies that $\Lambda =\frac{2}{P}$, where P is the period between two stripes, as shown in Figure 2.

o The critical contact line density Λ_c_, which is expressed as follows:

(4) $$\Lambda c=\frac{-\rho gV^{\frac{1}{3}}{(\tan\frac{\theta_{a}}{2}(3+{\left(\tan\frac{\theta_{a}}{2}\right)}^{2}))}^{2/3}}{({\left(36\pi \right)}^{1/3}\gamma \cos\left(\theta_{a,0}+\omega -90)\right)}$$

where ρ is the density, g is the gravity constant, V is the droplet volume, ω is the slope of the asperity, γ is the surface tension, θ_a_ is the advancing CA on the textured surface, and θ_a,0_ is the advancing CA on the smooth surface.

The Extrand model is described in more detail in the Supplementary Materials.

**Figure 3:**
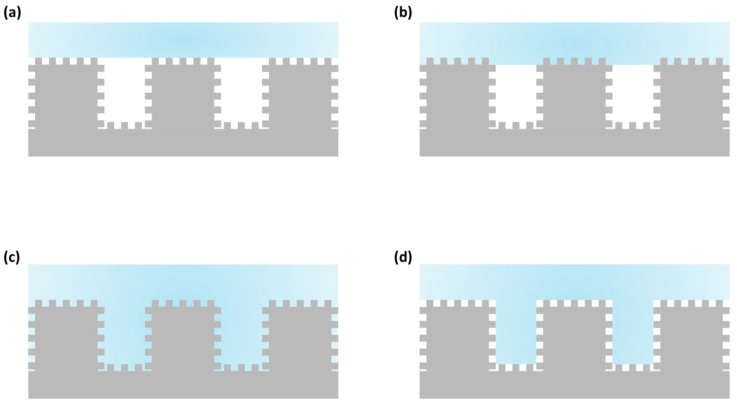
A schematic representation of the four configurations proposed by Rahmawan et al. [25]: (**a**) pure Cassie–Baxter state $\theta_{{CB}_{m}-{CB}_{n}}$; (**b**) pure Wenzel state $\theta_{W_{m}-W_{n}}$; (**c**) the Cassie–Wenzel mixed state $\theta_{{CB}_{m}-W_{n}}$; (**d**) the Wenzel–Cassie mixed state $\theta_{W_{m}-{CB}_{n}}$).

However, in some cases, more complex wettability phenomena occur, and the Wenzel and Cassie–Baxter models are insufficient to describe the wettability state. This is particularly the case with rose petals. As with the *M. peleides* butterfly, the rose petal has a double scale of roughness, resulting in exceptional wettability properties, which are also referred to as the “rose petal effect”. Indeed, when a droplet on the petal is in equilibrium on the petal with a high CA (approximately 150°), it adheres strongly to the surface and does not deform, even when the petal is tilted. This phenomenon occurs because the wettability of the micrometric scale occurs under the Cassie–Baxter conditions, whereas the wettability of the nanometric scale occurs under the Wenzel conditions. This wettability configuration leads to super adhesive properties and significant CA hysteresis (180°) [23]. Therefore, Rahmawan et al. [24] proposed four new configurations (Figure 3) combining the Wenzel and Cassie–Baxter states for surfaces with two scales of roughness. The apparent CA on these four configurations depends on the roughness and solid fraction of each scale, which are expressed as follows (for each configuration, m and n represent the micro- and nanostructures, respectively):

o The pure Cassie–Baxter state: both scales are wet in the Cassie–Baxter condition, and the associated apparent contact angle is:

(5) $$\cos\theta_{{CB}_{m}-{CB}_{n}}=\varphi_{s,m}\varphi_{s,n}(\cos\theta_{Y}+1)-1$$

o The pure Wenzel state: both scales are wet in the Wenzel condition, and the associated contact angle is:

(6) $$\cos\theta_{W_{m}-W_{n}}=r_{m}r_{n}(\cos\theta_{Y})$$

o The Cassie–Wenzel mixed state: the principal ridged stripes are wet in Cassie–Baxter and the cross-ribs are wet in Wenzel; the associated apparent contact angle is:

(7) $$\cos\theta_{{CB}_{m}-W_{n}}=r_{n}{(\varphi }_{s,m}(\cos\theta_{Y}+1)-1)$$

o The Wenzel–Cassie mixed state: the principal ridged stripes are wet in Wenzel and the cross-ribs are wet in Cassie–Baxter; the associated apparent contact angle is:

(8) $$\cos\theta_{W_{m}-{CB}_{n}}=r_{m}{(\varphi }_{s,n}(\cos\theta_{Y}+1)-1)$$

Due to their morphology, behavior, and environment, the wings of the *M. peleides* must be multifunctional. Numerous publications have focused mainly on the study of the structural patterns responsible for the iridescence of *Morpho* butterflies [9,10,11,12,13]. This study proposes a detailed analysis of the wettability properties of the butterfly *M. peleides* to identify the topographical and chemical features associated with wettability properties and distinguish them from those responsible for optical properties. The structures responsible for such wettability behavior are first investigated by scanning electron microscope (SEM) and atomic force microscopy (AFM) observations. Wettability properties are characterized through static CA and sliding angle (SA) measurements. Furthermore, the models of Extrand and Rahmawan et al. are used to describe the contribution to wettability of each scale of roughness of the *M. peleides* to better understand the wettability properties of this butterfly.

## 2. Materials and Methods

### 2.1. M. peleides Butterfly

*M. peleides* wings were purchased from a horticulturist (ETS JP Vesco, Visan, France). Each wing was used as received without any treatment or cleaning. As the CA measurements required a flat surface, wing pieces were cut to approximately 1.5 cm × 1.5 cm with a scalpel and taped onto a glass slide.

### 2.2. Topography Characterization

**Figure 4:**
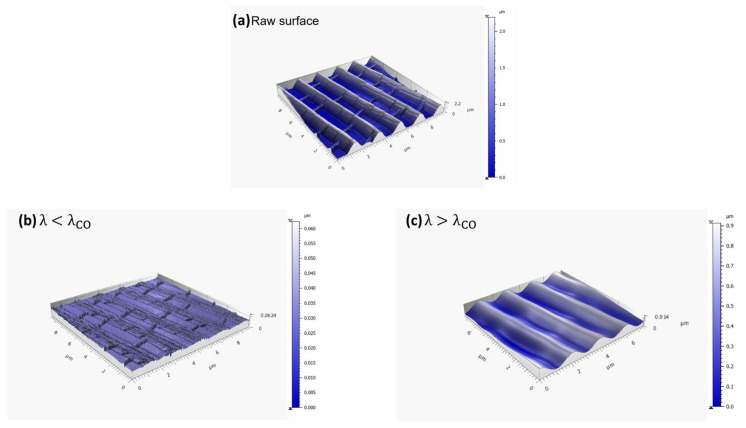
The Gaussian filter on the *M. peleides* wing: raw surface (**a**); the high-pass filter that suppresses large-scale components (**b**); and the low-pass filter that suppresses small-scale components (**c**).

*M. peleides* was purchased to characterize the wings’ structure. Both the dorsal and ventral wings were investigated using SEM (MIRA3, Tescan, Brno, Czech Republic) and AFM (Park Systems, Suwon, Korea). For the morphological characterization using SEM, the butterfly wings were first metalized. Therefore, a coating of a few nanometers of gold was made using a vacuum-sputtering metallizer. Regarding AFM, the corresponding images were analyzed using the MountainsMap software^®^ (version 7.4.8076) [25] (Digital Surf, Besançon, France) and the ISO-25178 roughness standard [26]. Due to the dual scale of roughness of the *M. peleides* butterfly structure, a Gaussian filter was used during AFM images analysis to investigate each scale separately [27]. The Gaussian filter depends on the cut-off spatial wavelength λ_CO_. Applying the Gaussian filter generated two distinct surfaces, as shown in Figure 4. The first surface includes wavelengths lower than the λ_CO_ value (Figure 4b) and the second comprises wavelengths exceeding the λ_CO_ value (Figure 4c). If λ = λ_CO_, the filter transmits 50% of its amplitude to each resulting surface. This characteristic underscores the importance of selecting a λ_CO_ value that is significantly distant from the wavelengths of the scales to separate it effectively. Once the appropriate scales are separated, the developed interfacial area ratio S_dr_ is calculated. Owing to this parameter, it is possible to determine the Wenzel parameter r as follows:

(9) $$r=S_{dr}+1$$

### 2.3. Wettability

To determine the wettability properties of the ventral and dorsal wings of the *M. peleides* butterfly, the CA and SA were measured using a goniometer (DSA 30, Kruss). For CA, a 3 µL droplet of distilled water was gently deposited onto the surface of interest. To ensure measurement reproducibility, five droplets were deposited onto the surface. The SA measurements were performed using the tilt method and droplets of distilled water of 3 µL. The surface was then tilted at 1°/s. During the acquisition process, five pictures/s were taken. The SA corresponds to the angle of inclination when the drop rolls off the surface. As shown in Figure 1, SA measurements were conducted in two directions: following the ⊕RO direction and the ⊖RO direction. As previously reported, five measurements were performed for each direction to ensure repeatability.

For both methods, CA and SA measurements were performed using Drop Shape Analysis software (version 1.92.1.1) and the tangent-2 method [28].

## 3. Results

### 3.1. Topography

#### 3.1.1. Topography of *M. peleides* Butterfly’s Wings

**Figure 5:**
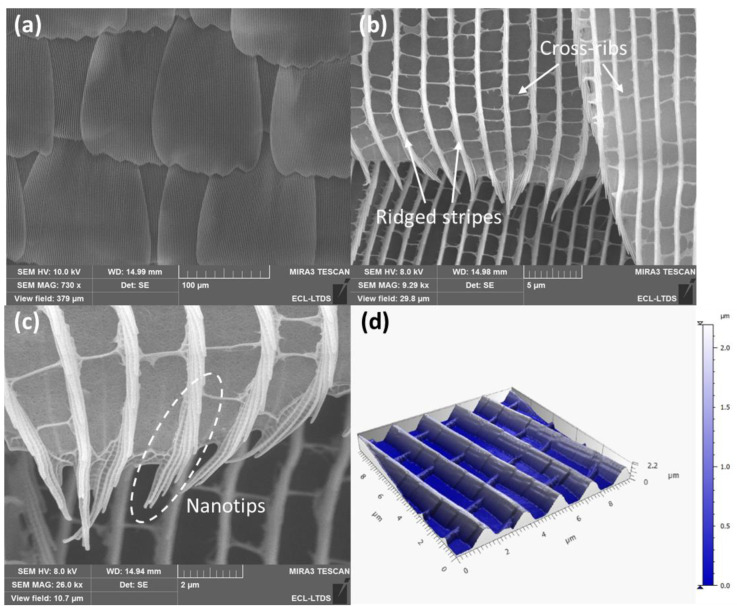
SEM (**a**–**c**) and AFM images (**d**) of micro–nanostructures of the *M. peleides’* dorsal wing.

To better understand the wettability properties of the *M. peleides* butterfly, the wing structure is first observed using SEM and AFM. These observations revealed that the dorsal wings of the *M. peleides* are covered with a periodic overlay of scales (Figure 5). The length of the scale ranged from 157 to 216 µm and the width ranged from 107 to 137 µm. Further magnified views show that each scale comprises numerous ridged stripes approximately 0.2 µm thick, spaced about 2 µm apart, and with a height of 1.71 µm. Moreover, each ridged stripe ends with nanotips (Figure 5c). These flexible nanotips are responsible for the anisotropic dynamic wettability.

**Figure 6:**
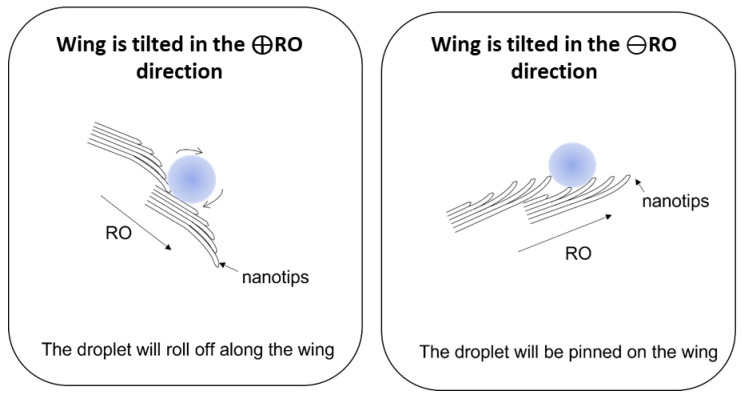
A schematic representation of the droplet adhesion along the ⊕RO and ⊖RO directions. This figure is inspired by Zheng et al.’s publication [14].

In 2007, Zheng et al. [14] reported this anisotropic behavior for the first time during their study of the *Morpho aega*. In fact, the *Morpho* butterfly can rearrange its micro–nanostructures according to the rolling direction. When the wings are tilted along the ⊕RO direction, the microscales are spatially separated from each other and the nanotips are oriented downward [14], as shown in Figure 6. In this case, the droplets roll off easily. Conversely, when the wings are tilted along the ⊖RO, i.e., inwards, the microscales take a close arrangement and the nanotips are raised [14] (Figure 6). In this configuration, the liquid/solid contact area is increased, and a droplet is pinned on the wing. As mentioned above, this phenomenon is closely linked to the survival of the *Morpho* butterflies. The droplet is intentionally immobilized to prevent it from wettability the butterfly’s body, which could add weight and hinder the butterfly’s ability to fly. Furthermore, this phenomenon allows the wings to be cleaned, because the legs are too small to reach the entire wing surface.

#### 3.1.2. Simplified Topography for Modeling

As discussed before, the wettability state of a textured surface can be predicted by using the Extrand model. Nevertheless, in order to calculate the parameters proposed by Extrand in his model (Λ, Λ_c_, and z_c_; see the Supplementary Materials), it is necessary to know the geometric parameters of the surface. Thus, the structure of the *M. peleides* wings is simplified and supposed as an array of stripes, as depicted in Figure 2. Thanks to the AFM images (Figure 5d) and MountainsMap software, the determination of each geometrical parameter of the texture was possible, and these are presented in Table 1.

### 3.2. Wettability Behavior

#### 3.2.1. Static Contact Angle of *M. peleides* Butterfly Wings

In 1996, Wagner et al. [17] reported a correlation between the wettability of butterfly wings and their wingspan. In their study, the authors demonstrated that butterflies with a large wingspan exhibited very unwettable wings and high particle removal. Regarding the *Morpho* butterfly, which has a large wingspan, its legs are too small to reach and clean the entire surface of its wings. Therefore, the *Morpho* butterfly is completely dependent on the self-cleaning properties of its wings.

As shown in Table 2, the dorsal wings of the *M. peleides* exhibit a CA of 136°. This value is in agreement with those reported by Wagner et al. [17] on butterflies of the same species. Furthermore, the CA measured on the ventral wings (130°) did not differ significantly from the CA obtained on the dorsal wings.

**Figure 7:**
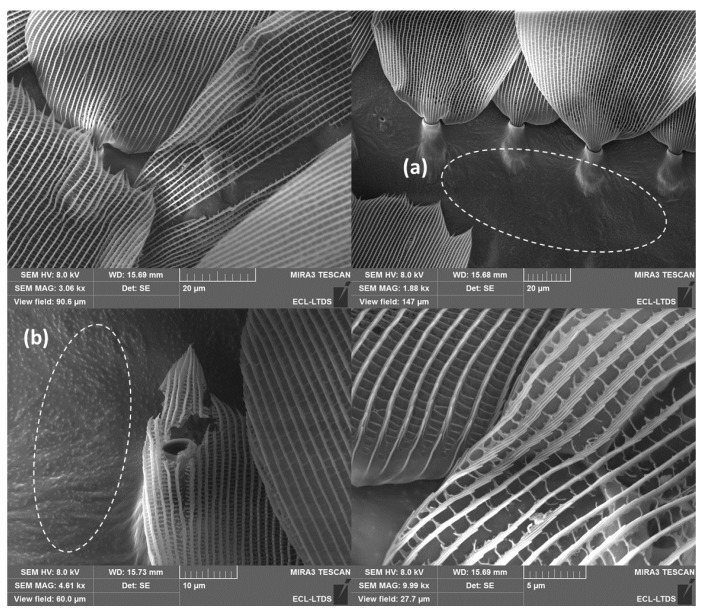
SEM images of the damaged dorsal wings of the *M. peleides* (gaps left by missing scales (**a**) and (**b**)).

Nevertheless, the wings of the *Morpho* butterfly are very fragile. As a result, they are often damaged at the end of their lives. Figure 7 shows the SEM images of the damaged micro–nanostructures or the loss of scales of the *M. peleides* wings. Thus, CA measurements were also performed on damaged wings (Table 2). In this study, the wing of *M. peleides* was damaged by pressing it between two glass slides. As expected, the CA was lower than that of the undamaged wings (108°). Figure 7 shows the SEM images of the damaged micro–nanostructures or the loss of scales on wings of *M. peleides*. This result highlights the importance of the wing structure in maintaining its high hydrophobicity and thus, its self-cleaning properties. In this absence of several scales or when the ridged stripes are damaged, the droplet can no longer remain suspended, and a transition from the Cassie–Baxter state to the Wenzel state may be observed. Nevertheless, given the dual scale of roughness of the wings, the wettability properties are far more complex and require the use of additional models to be explained. This will be the subject of the Discussions section.

#### 3.2.2. Sliding Angle of *Morpho peleides* Butterfly’s Wings

To understand the unique anisotropic dynamic wettability of the *M. peleides,* the SA was measured in two directions, as shown in Figure 1b. The measurements were conducted on the *M. peleides*’ dorsal wing, and the obtained results are presented in Table 3.

The SA along the ⊕RO direction is <10° (7°), indicating good self-cleaning ability. In contrast, against the RO direction, the SA is equal to 29°.

The results follow those reported by Zheng et al. [29], who found that the droplet rolls easily away from the body of the *Morpho sulkowskyi* butterfly at a tilted angle of approximately 10°. Along the ⊖RO direction, the droplet rolls off at a tilted angle of approximately 30°. Sun et al. [30] also measured the SA in various directions for 29 butterfly species. They also found an extremely significant difference in SA following the ⊕RO direction or ⊖RO direction.

Studies suggest that whether a droplet is pinned on or rolls off a superhydrophobic surface is attributed to a wettability state [31,32,33]. Indeed, if the droplet is pinned on, the contact surface area will increase, and the droplet will wet the texture in the Wenzel state, which may explain the high adhesion of the droplet. In contrast, along the ⊕RO direction, the contact surface area is smaller, and the droplet sits on top of asperities with air trapped at the submicrometer scales. This results in a nearly spherical droplet that rolls off on a texture with extremely low adhesion. However, as shown by Zheng et al.’s study [14], the pinning and rolling states are reversible. If the droplet had been in a Wenzel state as described above, it would have been impossible for the droplet to return to a Cassie–Baxter state. Therefore, when the wing is tilted along the ⊖RO direction, the droplet is in a metastable state. In other words, the droplet partially wets the surface with air trapped in the asperities.

Kusumaatmaja et al. [34] have also presented this metastable state in their study. They simulated the movement of a droplet on ratcheted superhydrophobic surfaces. This type of texture can be similar to the butterfly wing structure to provide an understanding of the droplet’s movement on butterfly wings.

## 4. Discussions

In order to understand the wettability properties of the *M. peleides* butterfly, the Extrand model was used. As discussed earlier, the Extrand model allows for the determination of the wettability state of textured surfaces. The geometric parameters of the *M. peleides* wings were determined previously. However, the Extrand parameters (Λ, Λ_c_, and z_c_; see the Supplementary Materials) depend on the apparent and advancing CAs on a smooth surface. Since these angles cannot be measured on a smooth *M. peleides* wing, several θ_a,0_ values are considered. If the model predicts the Cassie–Baxter state, the associated CA (θ_CB_) can be calculated using Equation (2). In the case of stripes, the area fraction of solid surfaces is given by the following Equation:

(10) $$\varphi_{s}=\frac{DP}{P^{2}}$$

If the model predicts the Wenzel state, Equation (1) is used. In this case, the roughness parameter r is determined using Equation (9).

**Figure 8:**
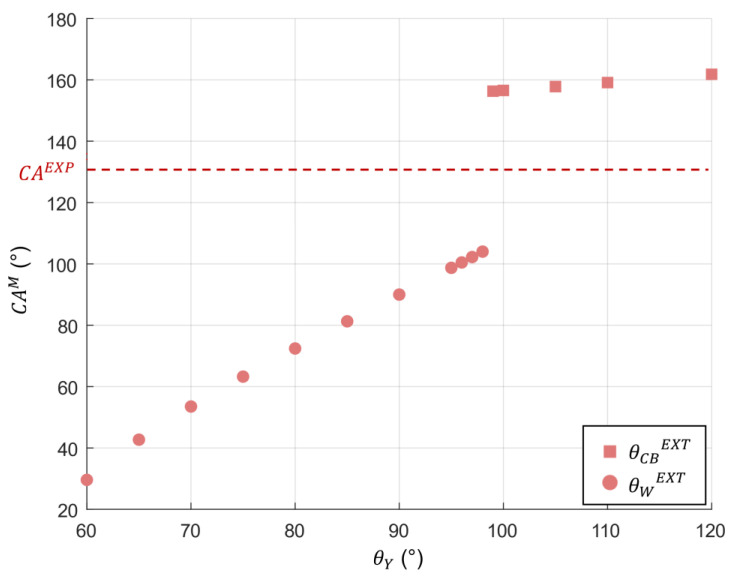
A graphical representation of the contact angle predicted by the Extrand model as a function of the CA on a smooth surface θ_Y_ (CA^M^ corresponds to the CA predicted by the model, in this case the Extrand model, θ_CB_^EXT^ corresponds to the predicted CA according to the Cassie–Baxter Equation, θ_W_^EXT^ corresponds to the predicted CA according to the Wenzel Equation, and CA^EXP^ corresponds to the CA experimentally obtained).

Figure 8 depicts the CAs predicted by the Extrand model as a function of different angles on smooth surface θ_Y_. The comparison between the CA^M^ and the CA obtained experimentally (CA^EXP^ = 136°) shows quite a high difference. This difference can be attributed to the fact that the Extrand model does not consider the dual scale of roughness. In fact, the wing structure is supposed to be an array of stipes; however, as shown in Figure 5, the scale comprises numerous ridged stripes connected with smaller cross-ribs. Therefore, it is possible that these cross-ribs have an influence on the wettability properties of the *M. peleides*.

Thus, the model proposed by Rahmawan et al. [24], that takes into consideration a dual scale of roughness, is used to determine which configuration accurately describes the wettability state of the *M. peleides*. The CA of each configuration depends on the roughness and solid fraction of each scale. In the case of the *M. peleides* butterfly, two scales are considered: the principal ridged stripes and the smaller cross-ribs. Based on the obtained AFM image (Figure 5), a cut-off wavelength was selected to separate each scale and determine the required parameters (S_dr_, P, and D). The corresponding roughness and solid fraction were then calculated according to Equations (9) and (10). Table 4 summarizes the values obtained.

**Figure 9:**
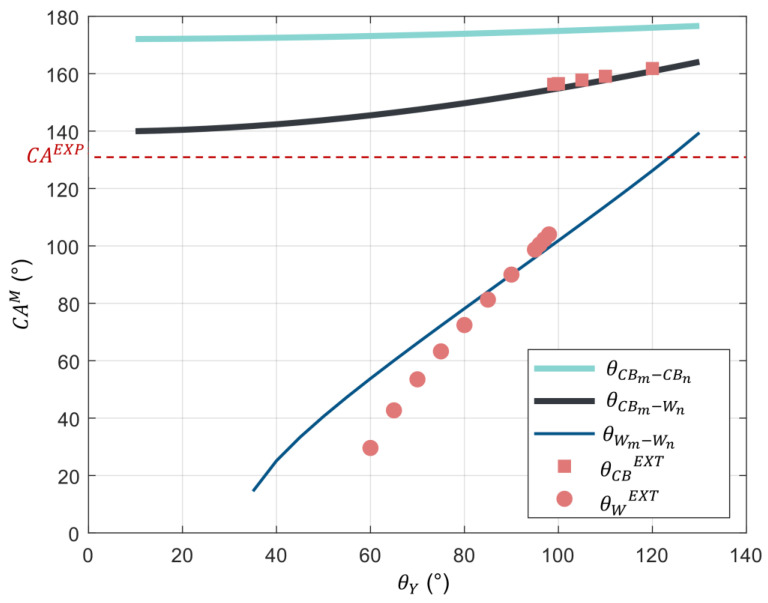
The evolution of contact angle predicted by the models (CA^M^) as a function of CA on a smooth surface θ_Y_ ($\theta_{{CB}_{m}-{CB}_{n}}$ corresponds to the pure Cassie–Baxter state, $\theta_{W_{m}-W_{n}}$ corresponds to the Cassie–Wenzel mixed state, $\theta_{W_{m}-W_{n}}$ corresponds to the pure Wenzel state, θ_CB_^EXT^ corresponds to the predicted CA by the Extrand model according to the Cassie–Baxter Equation, θ_W_^EXT^ corresponds to the predicted CA predicted by the Extrand model according to the Wenzel Equation, and CA^EXP^ corresponds to the CA experimentally obtained).

Equations (5)–(8) depend on the CA on a smooth surface (θ_Y_). However, as previously discussed, it is impossible to measure the CA on a smooth wing. Therefore, the CA of each configuration is plotted as a function of several θ_Y_ values (Figure 9). Using these curves, it is possible to determine which configuration accurately describes the wettability state of the *M. peleides* butterfly wings.

**Figure 10:**
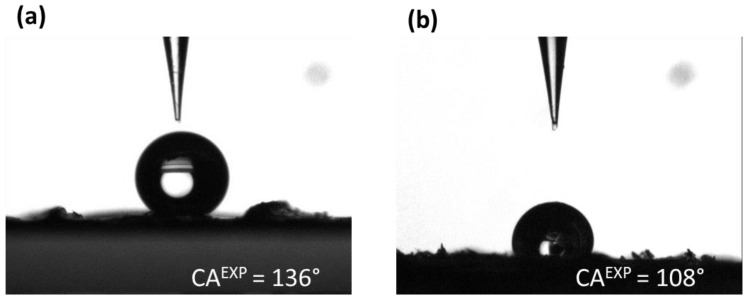
A 3 µL droplet of distilled water on an undamaged (**a**) and damaged (**b**) *M. peleides* dorsal wing.

**Figure 11:**
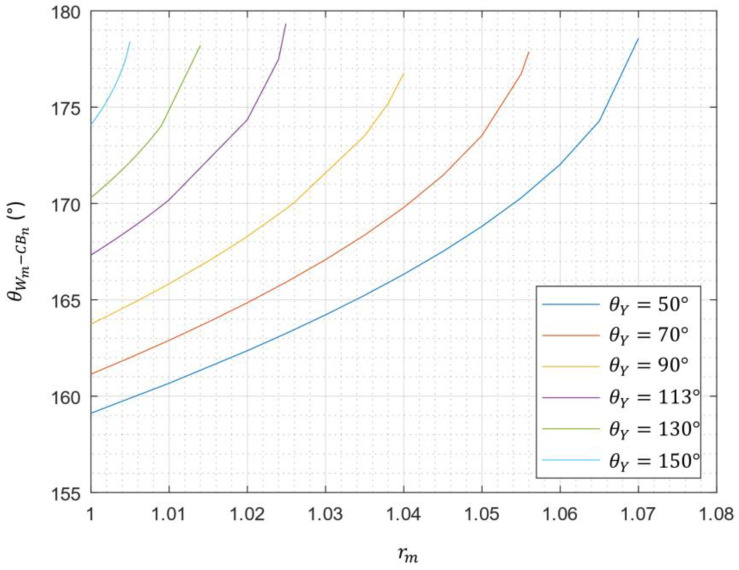
The evolution of predicted contact angle $\theta_{W_{m}-{CB}_{n}}$ as a function of r_m_ for φ_s_ = 0.04.

Regarding the pure Cassie–Baxter state ($\theta_{{CB}_{m}-{CB}_{n}}$), the calculated CA values are very far from the value obtained experimentally (CA^EXP^ = 136°). Therefore, the pure Cassie–Baxter state is directly eliminated. The pure Wenzel state $(\theta_{W_{m}-W_{n}}$), is also eliminated even if a CA of 136° is obtained for θ_Y_ = 127°. Indeed, as depicted in Figure 10a, the droplet has a quasi-spherical shape; therefore, it cannot wet the principal ridged stripes following the Wenzel state. For comparison, Figure 10b shows a droplet deposited on a damaged wing. As mentioned in Section 3.2.1., when a droplet is deposited on a damaged wing, a Cassie–Baxter to Wenzel transition is observed, resulting in a less spherical droplet. Concerning the Wenzel–Cassie mixed state ($\theta_{W_{m}-{CB}_{n}}$), the CAs were impossible to obtain because of excessive roughness. This is due to the limitations of the Wenzel state. As shown in Figure 11, for large roughness, it is impossible to calculate the associated $\theta_{W_{m}-{CB}_{n}}$, for a given θ_Y_ and solid fraction $\varphi_{s,n}$.

Therefore, the CAs predicted by the Cassie–Wenzel mixed-state configuration ($\theta_{{CB}_{m}-W_{n}}$) are the closest to the CA^EXP^. This result is consistent with what was mentioned in Section 3.2.2., the droplet partially wets the surfaces, and according to the model proposed by Rahmawan et al., the droplet wets the ridged stripes in the Cassie–Baxter state and the cross-ribs in the Wenzel state.

According to the graph presented in Figure 9, the CAs obtained with the Extrand model match those obtained for the Cassie–Wenzel mixed state and the pure Wenzel state. Thus, for such values of r_m_, r_n_, $\varphi_{s,m}$, and $\varphi_{s,n}$, the nanostructures appear to have more influence on the dynamic wettability than on the static wettability. Indeed, the results obtained with a model considering only a single scale of roughness are similar to those obtained with a model accounting for a dual scale of roughness.

**Figure 12:**
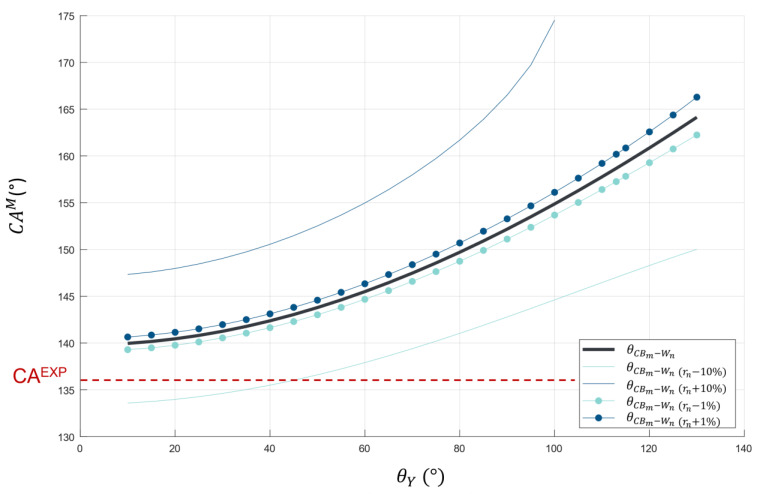
The evolution of the contact angle in the Cassie–Wenzel mixed-state configuration as a function of roughness deviations.

Nevertheless, the obtained curve for the Cassie–Wenzel mixed state ($\theta_{{CB}_{m}-W_{n}}$) does not consider potential errors in the roughness measurements. Figure 12 illustrates the evolution of the CA in the Cassie–Wenzel mixed-state configuration with various deviations in the roughness parameter r_n_. Regarding Figure 12, if r_n_ = r_n_−10%, a CA of 136° is reached for a θ_Y_ of approximately 40°. According to Elbaz et al. [35], it is well known that butterfly wings are made of chitinous structures. In another study, Greca et al. [36] measured a contact angle of 37° on pure chitin pulp film. Thus, this result supports the use of the model proposed by Rahmawan et al. to describe the wettability properties of the *M. peleides*. However, since the exact chemical composition of the *M. peleides* butterfly wings is unknown, this study does not allow for a definitive conclusion on an exact value of θ_Y_.

Nevertheless, this model confirms that the chemistry of butterfly wings has less influence than the wing structure with its dual-scale roughness on the wettability properties of the *M. peleides*. Regarding the corrected curve at r_n_ − 10% (Figure 12), if θ_Y_ is equal to 40° or 80, i.e., if θ_Y_ doubles, the associated predicted CA only increases by approximately 5°. On the contrary, according to Figure 9, for a same θ_Y_, the difference between the predicted CAs is much greater depending on the configuration. In other words, for the same chemistry, the influence of the topography is very significant. Furthermore, in the Supplementary Materials, CA measurements were performed on a dorsal wing coated with a thin layer of gold. A CA of 134° was measured, which is very close to the CA obtained on an uncoated wing. This result further confirms that the chemistry has less influence than topography on the wettability properties of the *M. peleides*.

## 5. Conclusions

This study proposes a detailed analysis of the wettability properties of the *M. peleides* wings to identify the contribution of different scales of topography and surface chemistry to these properties. In the case of the *M. peleides* butterfly, its micro–nanostructure imparts anisotropic dynamic wettability properties. In fact, when the wing was tilted along the RO direction, the SA was approximately 7°. Conversely, when the wing was tilted against the RO direction, the SA was approximately 29°. The dorsal wing of the *M. peleides* exhibited a CA of 136°. The relationship between the topography and wettability properties of the wings of the *M. peleides* butterfly was studied using the Extrand model and a hierarchical model proposed by Rahmawan et al. The results showed that the wettability properties of the *M. peleides* butterfly follow the Cassie–Wenzel mixed state. Moreover, under these conditions, the nanostructures appear to have greater influence on dynamic wettability than on static wettability. Additionally, compared to the contribution of the topography, the chemistry of the wing has very little influence on the wettability properties of the *M. peleides*.

Thus, given that the *M. peleides* wings are nearly superhydrophobic and exhibit unique dynamic wettability behavior, they can serve as a source of inspiration for designing surfaces with self-cleaning, anti-icing, anti-corrosive, water-repellent, and wear-resistant properties.

## Figures and Tables

**Table 1:** Geometric parameters of the *M. peleides* butterfly’s ridged stripes.

D (µm)	H (µm)	P (µm)	ꙍ (°)
0.20 ± 0.05	1.71 ± 0.03	2.01 ± 0.02	71.50 ± 0.75

**Table 2:** CA measurements on *M. peleides* wings.

Dorsal Wing	Ventral Wing	Damaged Wing
136 ± 1°	130 ± 1°	108 ± 1°

**Table 3:** SA measurements on the *M. peleides* wings.

⊖RO Direction	⊕RO Direction
29 ± 3°	7 ± 1°

**Table 4:** Roughness and solid fraction for each involved scale.

	λ_CO_ (μm)	S_dr_ (%)	r (According to Equation (1))	φs
Principal ridged stripes (m)	2.50	17.6	r_m_ = 1.176	$\varphi_{s,m}=$0.12
Cross-ribs (n)	0.08	0.521	r_n_ = 1.005	$\varphi_{s,n}=$0.04

## Data Availability

All data are available within the manuscript.

## Supplementary Materials

The following information can be downloaded at: https://www.mdpi.com/article/10.3390/biomimetics10020089/s1, Figure S1: Schematic representation of stripes seen from the side (a) and the top (b), Figure S2: 3 µL droplet of distilled water on a non-metallized and metallized *M. peleides* dorsal wing. References [18,19,20] have been cited in the Supplementary Materials.
