# Supplementary material for: How the Structure and Wettability Properties of Morpho peleides Butterfly Wings Can Be a Source of Inspiration
Source: Biomimetics (Basel). 2025 Feb 3;10(2):89. doi: 10.3390/biomimetics10020089 (PMC11853637; doi:10.3390/biomimetics10020089)
Supplement: Supplementary file 1 [file biomimetics-10-00089-s001.zip › biomimetics-3444494-supplementary.pdf]

Supplementary Material

How the Structure and Wettability Properties of *Morpho peleides* Butterfly Wings Can Be a Source of Inspiration

Louise Burdin [1,*], Anne-Catherine Brulez [1,2], Radoslaw Mazurczyk [3], Jean-Louis Leclercq [3] and Stéphane Benayoun [1]

[1] Ecole Centrale de Lyon, CNRS, ENTPE, LTDS, UMR5513, 69130 Ecully, France; anne-catherine.brulez@itech.fr (A.-C.B.); stephane.benayoun@ec-lyon.fr (S.B.)

[2] ITECH, 69130 Ecully, France

[3] Université de Lyon, CNRS, INSA Lyon, Ecole Centrale de Lyon, Université Claude Bernard Lyon 1, CPE Lyon, Institut des Nanotechnologies de Lyon, UMR5270, 69130 Ecully, France; radoslaw.mazurczyk@ec-lyon.fr (R.M.); jean-louis.leclercq@ec-lyon.fr (J.-L.L.)

* Correspondence: louise.burdin@ec-lyon.fr

1. The Extrand Model

In 2002, Extrand [1] was one of the first to propose a model to predict whether a liquid will remain suspended on the asperities of a surface (i.e., Cassie-Baxter state [2]) or will fill these asperities (i.e., Wenzel state [3]). This model is based on two new parameters:

- The contact line density Λ, defined using the following equation:

$$\Lambda = p\delta \qquad \text{(S1)}$$

where p is the asperity perimeter and δ is the area density of the asperities.

In the case of stripes, $p = 2P$ and $\delta = \frac{1}{P^2}$, which implies that $\Lambda = \frac{2}{P}$, where P is the period between two stripes as shown in Figure S1.

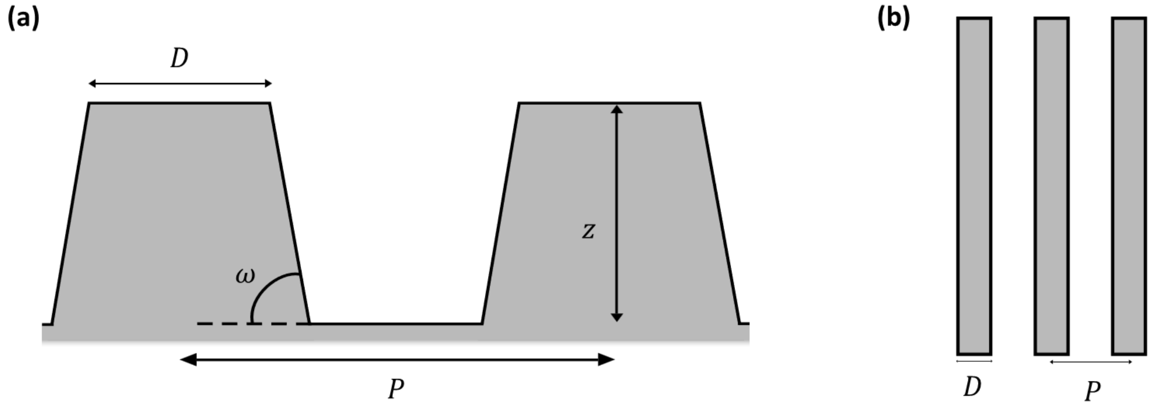

Figure S1. Schematic representation of stripes seen from the side (a) and the top (b).

- The critical contact line density Λ_c, which is expressed as follows:

$$\Lambda_c = \frac{-\rho g V^{\frac{1}{3}} \left(\tan\frac{\theta_a}{2}\left(3 + \left(\tan\frac{\theta_a}{2}\right)^2\right)\right)^{2/3}}{\left((36\,\pi)^{1/3} \gamma \cos(\theta_{a,0} + \omega - 90)\right)} \qquad \text{(S2)}$$

Where ρ is the density, g is the gravity constant, V is the droplet volume, ω is the slope of the asperity, γ is the surface tension, θ_a is the advancing contact angle (CA) on the textured surface, and $\theta_{a,0}$ is the advancing CA on the smooth surface.

If $\Lambda > \Lambda_c > 0$, the liquid droplet is held above the textures and the wettability state follows the Cassie-Baxter approach. Otherwise, if $\Lambda c < 0$ or $\Lambda < \Lambda c$, the system remains in the Wenzel model.

However, this first condition ($\Lambda > \Lambda_c > 0$) is necessary but not sufficient. The droplet meniscus between two asperities also plays an important role. Indeed, if this meniscus is larger than the texture height, the droplet fills the entire asperity leading to a state transition from Cassie-Baxter to Wenzel. Therefore, the second condition is as follows: $z > z_c$, where z_c is the height meniscus and is defined as follows:

$$z_c = b\ \tan\left(\frac{\theta_{a,0} + \omega - 180}{2}\right) \quad \text{(S3)}$$

where 2b is the distance between the asperities. In the case of stripes,

$$2b = P - D \quad \text{(S4)}$$

where D is the diameter of the texture.

2. Contact Angle Measurements on a Metallized Wing

In order to study the influence of wing chemistry on the wettability properties of *Morpho peleides* (*M. peleides*), a dorsal wing was metallized (SC7640 auto/manual high resolution sputter coater, Polaron, Quorum Technologies) for 200s to coat it with a thin gold layer a few nanometers thick. A goniometer (DSA 30, Kruss) was used to measure the CA on the metallized wing. Five droplets of 3μL of distilled water were deposited at different positions on the wing. The final CA corresponds to the average of the five measurements values. Figure S2 presents the CAs measured on both non-metallized and metallized wings. The images were obtained using the Drop Shape Analysis software and analyzed with the ImageJ software using the Drop analysis – DropSnake function. The CA was calculated by averaging the left and right CAs.

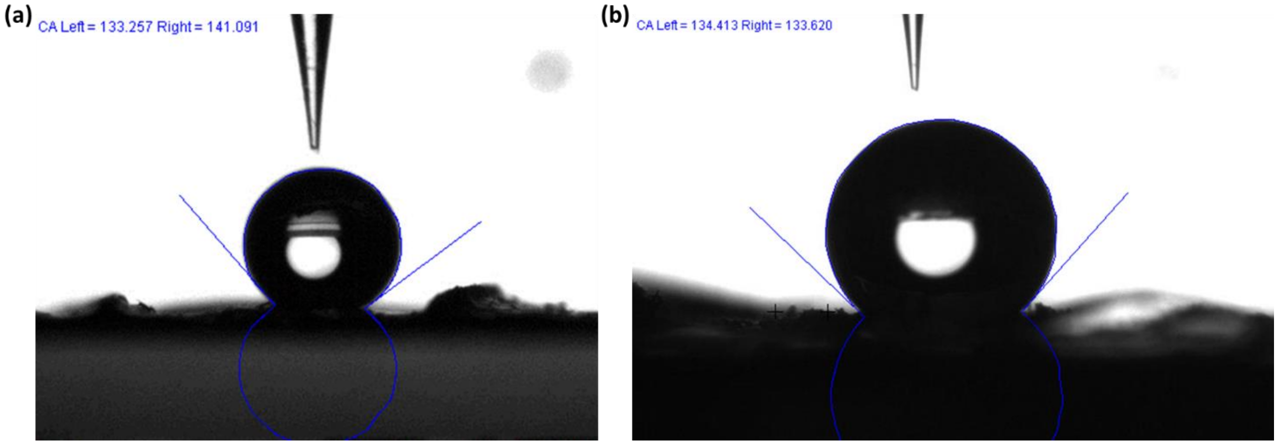

Figure S2. 3μL droplet of distilled water on a non-metallized and metallized *M. peleides* dorsal wing.

For the metallized wing, a final CA of 134±1° was obtained, which is very close to the CA of the non-metallized wing (136±1°). This result further confirms that the chemistry of the wing has less influence on the wettability properties of the *M. peleides* compared to the wing's topography.

References

1. Extrand, C.W. Criteria for Ultralyophobic Surfaces. *Langmuir* **2004**, *20*, 5013–5018. https://doi.org/10.1021/la036481s.
2. Cassie, A.B.D.; Baxter, S. Wettability of porous surfaces. *Trans. Faraday Soc.* **1944**, *40*, 546. https://doi.org/10.1039/tf9444000546.
3. Wenzel, R.N. Resistance of Solid Surfaces to Wetting by Water. *Ind. Eng. Chem.* **1936**, *28*, 988–994. https://doi.org/10.1021/ie50320a024.
